# Aerobic exercise training attenuates the deleterious effects of walker-256 cancer: effects on physical capacity, cachexia, and cardiac mass

**DOI:** 10.3389/fonc.2026.1734435

**Published:** 2026-06-29

**Authors:** Luis F. Rodrigues, Bruno R. A. Pelozin, Edilamar M. Oliveira, Tiago Fernandes

**Affiliations:** Laboratory of Biochemistry and Molecular Biology of Exercise, School of Physical Education and Sport, University of São Paulo, São Paulo, Brazil

**Keywords:** aerobic training, cachexia, cardiac remodeling, cardio oncology, walker-256 tumor

## Abstract

**Background:**

Aerobic exercise training (AET) is widely recognized for its preventive and therapeutic efficacy in chronic non-communicable diseases. Although studies have shown that AET delays tumor growth and cachexia in Walker-256 tumor-bearing rats, little is known about the impact of AET on cardiac phenotypes in different cancer models. Therefore, the aim of this study was to investigate cancer cachexia-induced cardiac changes in Walker-256 tumor and to analyze the therapeutic effects of AET on these changes.

**Methods:**

Wistar (n= 24) male rats were assigned into 3 groups: Wistar control (WC), control tumor (WCT), and tumor trained (WTT). Walker-256 tumor cells were subcutaneously injected into cancer groups to establish a robust cancer cachexia model. Swimming exercise training lasted 60 min, 5×/week/6 weeks, with 5% body weight workload. We assessed: exercise tolerance, hemodynamic parameters, tumor growth and cachexia, skeletal muscle and left ventricular mass by ratio of tissue weight/tibia length. Cardiac morphology and function were evaluated by echocardiography.

**Results:**

Walker-256 tumor induced exercise intolerance in the WCT group (267.65 ± 53.55 m; p < 0.05) compared to the WC group (455.77 ± 50.22 m). In contrast, AET prevented the exercise intolerance in the WTT group (578.63 ± 66.84 m) compared to control. WTT group showed a reduction in tumor weight (25.08 ± 4.05 g; p < 0.001) and the percentage of cachexia (7.83 ± 1.75%; p < 0.05) compared to the WCT group (47.08 ± 6 g/13.75 ± 1.71%), indicating a greater evolution of tumor growth and body weight loss in relation to trained group. Skeletal muscle wasting (soleus, gastrocnemius, plantaris and tibial anterior) was observed in cancer cachexia compared to control. In this way, Walker-256 tumor exhibited cardiac remodeling characterized by cardiac atrophy. AET reversed soleus and plantaris atrophy and led to significant anti-cardiac remodeling evaluated by LV mass in Walker-256 tumor.

**Conclusion:**

Our data provides a robust model of cancer cachexia, as well as highlights the effects of AET as a preventive strategy for mitigating the exercise intolerance, tumor growth, cachectic status, and the cardiac damage in Walker- 256 tumor.

## Introduction

Cardiovascular disease and cancer are the main causes of death in the world with 19 and 9.7 million deaths, respectively (1, 2). The incidence of cancer is expected to increase by up to 77% over the next two decades. Furthermore, it is estimated that by 2050, there will be around 35 million new cases worldwide, which represents a problem for health systems (2).

Cancer refers to a group of more than 100 diseases characterized by common alterations in the cell cycle, leading to disorganized cell growth and proliferation. Its development results from genetic changes, including increased expression of oncogenes and reduced activity of tumor suppressor genes, which together promote tumor growth (3, 4). Cancer results in several comorbidities, including cachexia, a complex multifactorial syndrome characterized by the loss of skeletal muscle mass (with or without loss of fat mass) that cannot be reversed by conventional nutritional support, which is of particular interest due to its high prevalence (present in 50–80% of patients with cancer) and high mortality (up to 30% of all cancer-related deaths) (5, 6). Thus, cancer cachexia is directly related to a progressive functional disability accompanied by exercise intolerance, breathlessness, fatigue, poor responses to chemotherapy, and decreased survival, which are clinical signs of heart failure (5–7). Importantly, heart failure is present in a substantial proportion of cancer cachexia-induced deaths (8–12).

As a multiorgan syndrome, cancer cachexia has been described to promote cardiac damage in animals and patients with cancer (8–11, 13). It was shown that patients with cancer may present cardiac dysfunction during a treadmill exercise test (13, 14). Indeed, cardiac atrophy has been reported in patients with cancer who died from cancer cachexia and had a significant reduction in heart weight (25.6%) and left ventricular (LV) wall thickness (12.1%) compared with control patients (8). A reduced LV mass, associated with thinning of the LV walls and decreased stroke volume, has been observed in patients with cancer-related cardiac atrophy. This condition was accompanied by an increase in heart rate (possibly as a compensatory mechanism) (15). Among the atrophy-mediated processes, programmed cell death, or apoptosis, with a general loss of healthy myocardial tissue, is reported to be increased in several cardiomyopathies (9–11, 16). Similarly, emerging studies have demonstrated cancer-induced cardiac dysfunction and remodeling in rodent models (11, 16, 17). In this sense, recent findings have demonstrated that Walker-256 tumor growth, as an experimental model of cachexia, represents an important effect on lean body mass wasting and heart remodeling, suggesting the presence of cardiac cachexia in these animals (10). Cardiac atrophy appears to occur in parallel with an increase in interstitial fibrosis, with implications mainly associated with decreased contractility and impaired diastolic function (11). Walker-256 tumor-bearing rats were first observed in 1928 by researcher George Walker, in the region of mammary gland. This model mimics many of the alterations induced by human tumors and has been used in several studies due to easy manipulation and injection (10, 18–24). Additionally, cancer therapy may result in adverse effects to the cardiovascular system such as cardiotoxicity and LV dysfunction (25–27). Although the negative actions of the cancer treatment on the cardiovascular system are quite evident, little is known about the damage caused by cancer on the heart before treatment in the setting of cancer cachexia. Therefore, identifying strategies to understand and treat cardiac alterations in cancer cachexia should be considered.

Aerobic exercise training (AET) has been shown to reduce cardiotoxicity often resulting from chemotherapy treatment, fatigue, and pro-inflammatory markers (28–30). Over the last years, studies have shown that AET delayed tumor growth and cancer cachexia in Walker 256 tumor-bearing rats (18, 22). AET effects in this model also include 1) an increase in aerobic consumption of substrates and prevention of glucose and glutamine metabolism impairment in immune cells (i.g. lymphocytes and macrophages) associated with an increased survival (31, 32), a decrease in tumor growth (22, 23, 31, 32), a prevention of body mass losses (22–24, 31, 33), and a decrease in skeletal muscle degradation process associated with an increase in skeletal muscle myosin content (34). Studies have shown the benefits of AET on cardiac function and structure in different cancer models (11, 17, 32). However, no study to date has been conducted evaluating the effect of AET on cardiac phenotype in Walker-256 tumor model. Therefore, this study aimed to investigate cancer cachexia-induced cardiac changes in Walker-256 tumor and to analyze the therapeutic effects of AET on these changes.

## Materials and methods

### Animals and experimental groups

All procedures and protocols used were in accordance with the local ethics committee of the University of São Paulo (No. 2021/01) and were conducted in accordance with the Guide for the Care and Use of Laboratory Animals, published by the US National Institutes of Health (NIH Publication No. 85-23, revised 1996). Three-week old male (100–120 g, n = 24) Wistar rats were used. The animals were weighed weekly and housed three to five per cage at a controlled room temperature (22 ± 2 °C) with a 12-h dark-light cycle and fed standard rat chow with access to water *ad libitum*. The rats were randomly assigned into three experimental groups: Wistar control (WC, n = 8), control tumor (WCT, n = 8), and tumor trained (WTT, n = 8).

### Exercise training protocol

**Figure 1:**
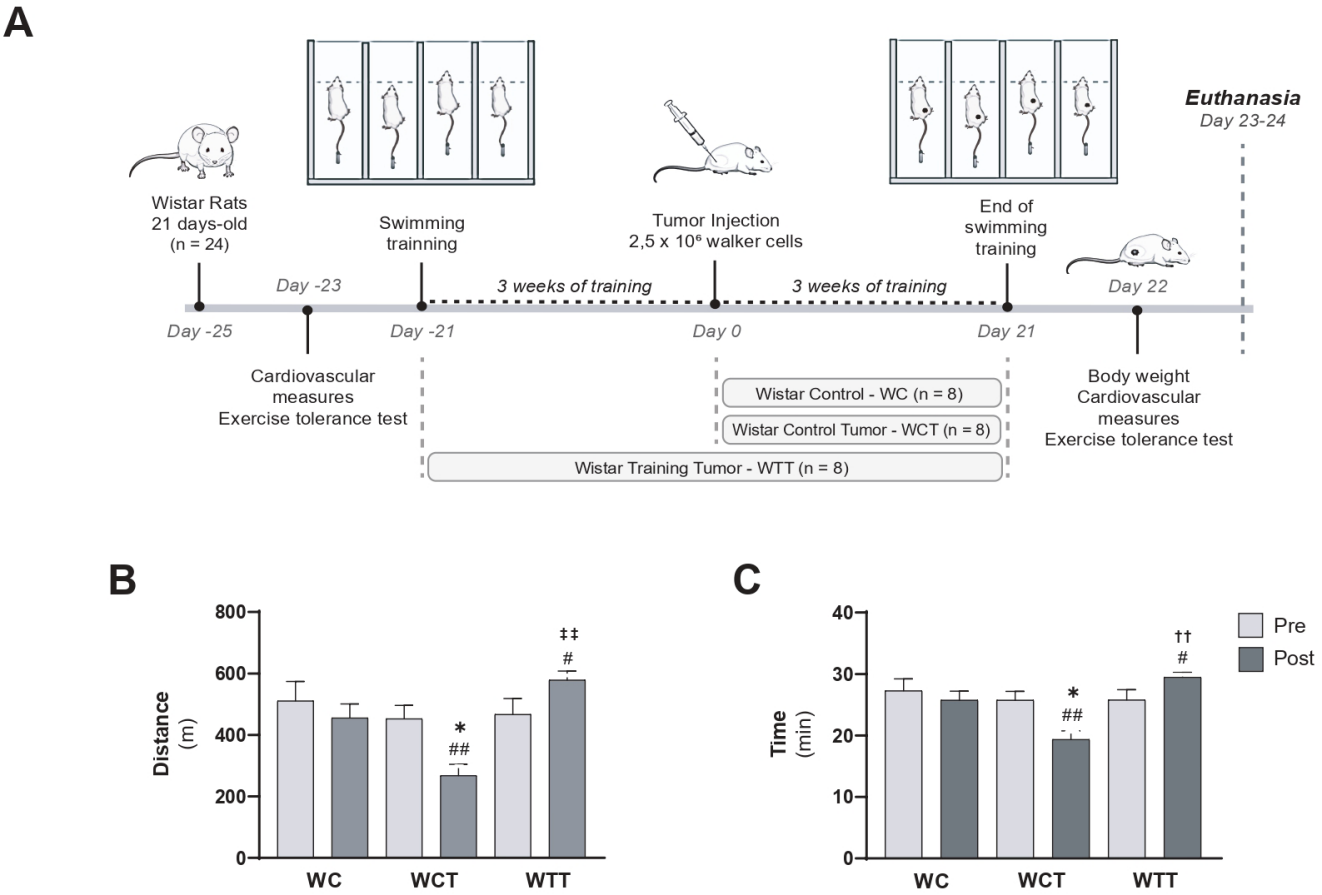
Schematic panel illustrating the study design: male Wistar rats were submitted to six weeks of aerobic exercise training. Twenty-one days later Walker-256 tumor cells were injected subcutaneously into the right flank of each rat, which were randomly assigned into Wistar Control, Wistar Control Tumor, and Wistar Trained Tumor. Tumor volume, body weight, and exercise tolerance were measured during the experimental period **(A)**. Aerobic exercise training prevented exercise intolerance induced by Walker-256 cancer assessed by the distance **(B)** and time **(C)**. Groups: WC indicates Wistar Control (n = 8), WCT indicates Wistar Control Tumor (n = 8), and WTT indicates Wistar Trained Tumor (n = 8). Results are expressed as mean ± SEM. Statistical analysis: One-way ANOVA, Tukey’s *post hoc* test. Significant different vs. # indicates the difference between pre-test and post-test. ^#^p < 0.05; ^##^p < 0.001. *WC, p < 0.05; ^††^WCT, p < 0.001; ^‡‡^WCT, p < 0.0001.

The protocol consisted of swimming sessions of 60-min duration, 5 times a week, for 6 weeks with a 5% caudal body weight (BW) workload (35). The animals trained three weeks before inoculation and three weeks after inoculation. All training had a 24-h interval between sessions; **Figure 1A** illustrates the training protocol groups. To allow progressive adaptation to the exercise protocol, animals underwent one week of familiarization during which swimming sessions were performed without any external load. Following this initial period, the workload was gradually increased by 1% of the animal’s BW every two days until reaching the target load of 5%. The workload was individually adjusted each week based on the animals’ weekly BW measurements to ensure appropriate training intensity throughout the protocol. The target duration and workload were maintained for all WTT animals until the end of the protocol.

All training was conducted in a swimming apparatus specially designed to allow individual exercise training of rats, a heating system kept the water temperature between 30 °C and 32 °C, and swimming sessions were carried out from 8:30 to 9:30 AM. Control animals (WC and WCT groups) were also placed in the swimming apparatus for 10 min, twice a week, without any external load and without progression in duration or intensity, solely to account for the effects of water exposure and handling. Importantly, this brief, unloaded water exposure was not intended to constitute an exercise training protocol and does not represent AET. Only the WTT group underwent structured AET (60 min/session, 5×/week, 6 weeks, with 5% body weight workload).

### Inoculation of walker 256 tumor cells

Walker-256 tumor is a mammalian adenocarcinoma widely used as an experimental model to induce cachexia and consequent muscle wasting in rats. Tumor implantation was performed according to the protocol described by Padilha et al. (24). The Walker-256 cell line was kindly provided by Professor Maria Cristina Cintra Gomes-Marcondes.

Briefly, tumor cells were obtained from ascitic tumors developed in host animals after intraperitoneal inoculation. For these ascitic cell passages, six additional young rats (3–4 weeks old) were used exclusively for the generation of ascitic tumors and expansion of the Walker-256 cell line; these animals were not included in the experimental groups. Two sequential passages were performed before subcutaneous inoculation, and the resulting cells were stored at −80 °C until use. In the first passage, 0.8 mL of ascitic fluid containing tumor cells was mixed with 0.2 mL of saline and injected intraperitoneally; ascitic fluid was collected approximately 6–7 days later. The second passage followed the same procedure; however, ascitic fluid was harvested after about 3 days because the tumor cells were more aggressive at this stage, which reduced the animals’ lifespan by approximately 3 days.

Cell viability was assessed using the trypan blue exclusion method with a Neubauer counting chamber. For experimental groups WTT and WCT, 2.5 × 10^6^ viable Walker-256 carcinoma cells suspended in 0.5 mL of PBS (pH 7.4) were injected subcutaneously into the right flank of each rat. Control animals received 0.5 mL of PBS (pH 7.4) in the same region. All procedures complied with the guidelines of the United Kingdom Co-ordinating Committee on Cancer Research (UKCCCR, 1998) for the welfare of animals in experimental neoplasia.

### Euthanasia and tissue preparation

At the end of the experimental period, the animals were euthanized by decapitation following prior anesthesia with 3–5% isoflurane administered via inhalation. After euthanasia, kidney, liver, spleen, lung, retroperitoneal fat, periepididymal fat, heart and skeletal muscle were promptly collected. The heart was dissected to isolate the LV, right ventricle (RV), and atria. From the right hindlimb, skeletal muscles including the soleus, gastrocnemius, tibialis anterior (TA), and plantaris were excised. Additionally, the tumor mass was removed from the right flank region of each animal. All samples were weighed, snap-frozen in liquid nitrogen, and stored at –80 °C for further analysis.

### Evaluation of tumor growth and cachexia

Evaluation of tumor growth was realized every other day after tumor cells were injected, using a pachymeter. The largest and smallest diameter of tumor was measured as previously described by Bergers et al. (33). The values obtained were used in the formula for estimated tumor volume: V = 0,52 X (larger diameter) X (smaller diameter)^2^.

For analysis of cachexia, after 21 days each tumor mass was removed and weighed, and total carcass weight (total body weight minus the tumor weight) was measured. Considering initial and final BW of the rats with tumor, tumor weight and body weight gain in control groups, using the following equation: BWL (%) = 100 x (iBW – fBW + tm + BWg)/(iBW + BWg). Where iBW is initial BW (g), fBW is final BW (g) of rats with tumor, tm is tumor mass (g) and BWg is BW gain (g) of rats without tumor. The rats were cachectic when BWL was higher than 10% (20, 21, 23, 36).

### Cardiovascular measurements

Resting systolic blood pressure (SBP), diastolic (DBP), medium (MBP) and HR were measured in conscious rats, with the use of a computerized tail cuff system (IITC Life Science, Woodland Hills, CA, USA). Rats were acclimatized to the apparatus during daily sessions held over 4 days, 1 week before starting the experimental period. Cardiac hemodynamics were assessed in pre- and post-tests during the AET period.

### Exercise tolerance test

Exercise capacity was evaluated using a graded treadmill exercise protocol, with total running distance and test duration used as outcome measures. Prior to the test, animals underwent a one week adaptation period consisting of daily 10 min sessions on a motorized treadmill at a speed of 12 m/min and 0% incline. This period was used solely for familiarization and was not considered part of an exercise training protocol (AVS, Model 1, AVS Projetos, SP, Brazil). On the day of testing, animals were placed on the treadmill and allowed to acclimate for at least 30 min before initiating the protocol. The exercise test began at a speed of 6 m/min with no incline, and speed was increased by 3 m/min every 3 minutes until the point of exhaustion. Exhaustion was defined as the animal’s inability to maintain the required pace despite gentle mechanical stimulation, which consisted of light tactile stimulation with a soft brush applied to the hindquarters and, when needed, a gentle tap on the treadmill surface behind the animal. No electrical or painful stimuli were used. To verify the effectiveness of exercise training, we addressed the exercise tolerance through pre- and post-tests during the AET period.

### Measurement of cardiac hypertrophy and function

Cardiac hypertrophy was evaluated by the wet weight of the total heart and LV corrected by tibia length (mg/mm) and by echocardiography (LV mass) in mg. The echocardiography procedure followed the guidelines recommended by the American Society of Echocardiography (37). Transthoracic echocardiography was performed after the training period based on the average of three consecutive cardiac cycles. The equipment used was a VEVO 2.100 (Visual Sonics Inc, Toronto, Canada) echocardiographer combined with a transducer (MS550D) of 40 MHz. Images were obtained with the transducer placed on the animal’s shaved chest (lateral recumbent position). To optimize imaging, a transmission gel was used between the transducer and the animal’s chest (general imaging gel; ATL, Reedsville, PA). Animals were scanned from below at a depth of 2 cm with the focus optimized at 1 cm. Rats were anesthetized in isoflurane (2% with 1 L/min 100% O_2_). Wall thickness and LV dimensions were obtained from a short-axis view at the level of the papillary muscle. LV mass was calculated using the following formula, assuming a spherical LV geometry and previously validated in rats: LV mass = 1.047 x [(LVID;d+ LVPW;d+ IVS;d)^3^ – LVID;d^3^], where 1.047 is the specific gravity of muscle, LVID;d is the internal diameter of the left ventricle in diastole, LVPW;d is the posterior wall thickness of the left ventricle during diastole, and IVS;d is the interventricular septal thickness during diastole. The cardiac function was estimated by the ejection fraction (EF) as follows: EF (%) = [(LVID;d - LVID;s)/LVID;d x100], where the LVID;d is the internal diameter of the left ventricle in diastole, and LVID;s is the internal diameter of the left ventricle in systole. Two-dimensionally guided pulsed Doppler recordings of LV transmitral flow were obtained from the apical 4-chamber view. Corroborating this, cardiac function was also assessed using the following parameters: fractional shortening (FS), cardiac output (CO), A wave (MV A), E wave (MV E), E wave deceleration (MV Decel), and early-to-late diastolic mitral inflow ratio (E/A ratio). Isovolumic relaxation time (IVRT) was taken as the time from aortic valve closure to the onset of mitral flow.

### Statistical analysis

The results are expressed as mean ± standard error of the mean (SEM). Data normality was assessed using the Shapiro–Wilk test. For variables measured at a single time point, one-way ANOVA followed by Tukey’s *post hoc* test was used. For longitudinal data (hemodynamic parameters and maximal exercise tolerance assessed pre- and post-intervention), a two-way repeated measures ANOVA was applied, with ‘group’ (WC, WCT, WTT) as the between subject factor and ‘time’ (pre, post) as the within subject factor, followed by Tukey’s *post hoc* test when appropriate. Statistical analyses were performed using GraphPad Prism software (Version 8.01, La Jolla, CA, United States). A p value < 0.05 was considered statistically significant.

## Results

### Effect of AET on exercise tolerance in walker-256 tumor-bearing rats

There were no differences in exercise tolerance for pre-protocol test among all groups, regarding distance (**Figure 1B**) and time (**Figure 1C**). In **Figure 1B**, in the post-protocol exercise tolerance test, there was no difference between the WC and WTT groups; however, the WCT group showed a reduction in the running distance (267.65 ± 53.55 m; p < 0.05) compared to the control group WC (455.77 ± 50.22 m). When comparing the results of the pre- and post-test of the WCT group, there was a reduction in the distance (pre-test: 452.57 ± 43.47 m; post-test: 267.65 ± 53.55 m; p < 0.01). The WTT group showed a significant increase in the post-test (578.63 ± 66.84 m; p < 0.0001) compared to the WCT group. In comparison between pre- and post-test, the WTT group showed an improvement to the final running distance (pre-test: 467.12 ± 51.11 m; p < 0.05). Regarding the time, **Figure 1C** showed similar results between groups in pre-test, whereas in post-test WCT group (19.34 ± 3.48 min; p < 0.05) have reduced time compared to the WC group (25.77 ± 1.63 min). Compared to pre- and post-test of the WCT group, there was a reduction in the time (pre-test: 25.76 ± 1.40 min; p < 0.01). At the end of the protocol, the WTT group showed an increase in test time (29.48 ± 3.20 min; p < 0.001) when compared to the WCT group. In the WTT group, test duration increased from 25.80 ± 1.64 min at pre-test to 29.48 ± 3.20 min at post-test (p < 0.05).

### Effect of AET on hemodynamic parameters in walker-256 tumor-bearing rats

There was no statistical difference between hemodynamic parameters (**Table 1**). SBP, DBP, MBP and HR were not affected by Walker-256 tumor and AET conditions.

**Table 1:** Hemodynamic parameters.

Hemodynamic parameters	Time	WC	WCT	WTT
SBP	Pre	134.41 ± 1.26	132.83 ± 1.07	135.66 ± 1.47
	Post	137.70 ± 0.60	135.66 ± 1.41	137.62 ± 0.57
MBP	Pre	102.29 ± 3.60	98 ± 1.93	100.45 ± 4.23
	Post	100.45 ± 1.28	96.83 ± 3.73	104.95 ± 3.49
DBP	Pre	87.04 ± 4.89	84.54 ± 2.09	83.62 ± 4.65
	Post	78.83 ± 1.90	77.04 ± 3.90	87.70 ± 3.53
HR	Pre	444.75 ± 18.99	452.62 ± 12.43	429.16 ± 13.15
	Post	430.33 ± 13.32	450.62 ± 21.17	462.47 ± 10.40

Systolic blood pressure (SBP), mean blood pressure (MBP), Diastolic blood pressure (DBP), Blood pressure are expressed in millimeters of mercury (mmHg), Heart rate (HR) is expressed in beats per minutes (bpm). Groups: WC indicates Wistar Control (n = 8); WCT Wistar Control Tumor (n = 8); and WTT, Wistar Trained Tumor (n = 8). Results are expressed as mean ± SEM. Statistical analysis: One-way ANOVA, Tukey’s post hoc test.

### Effect of AET on body weight, tumor progression, and cachexia in walker-256 tumor-bearing rats

**Figure 2:**
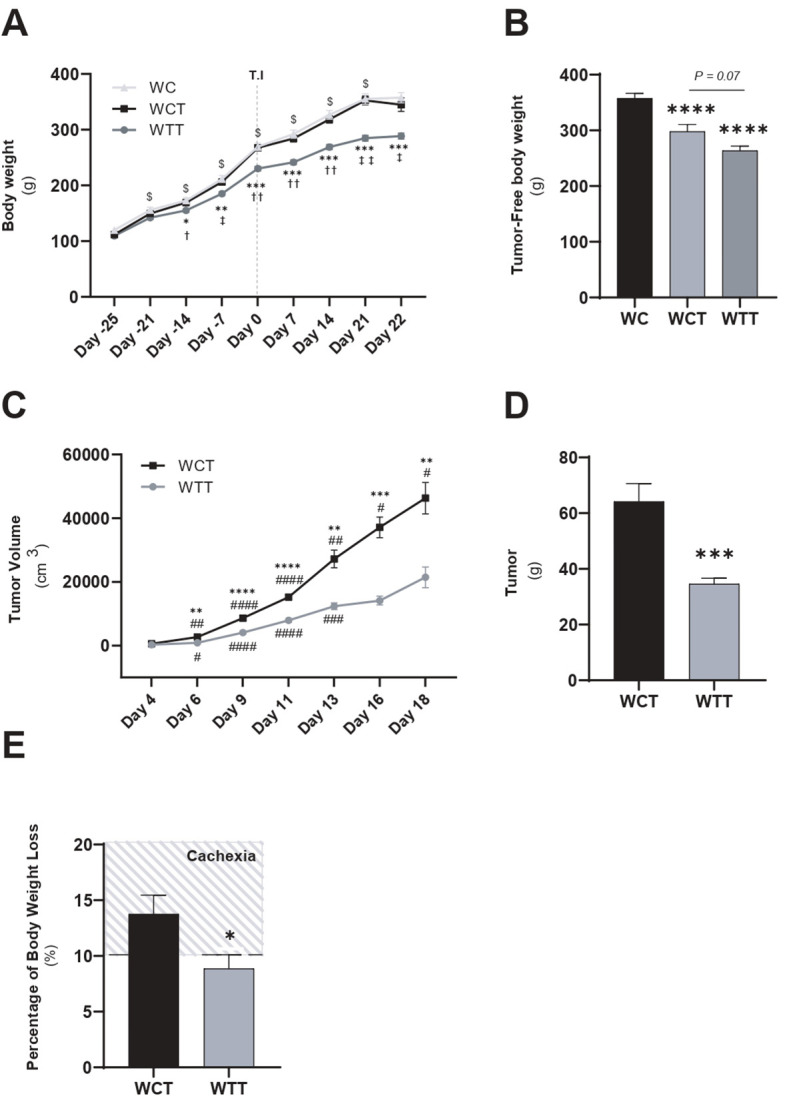
Aerobic exercise training-induced reduction tumor progression and cachexia. Body weight **(A)**, tumor-free body weight **(B)**, tumor volume progression **(C)**, tumor weight **(D)**, and percentage of body weight loss **(E)**. Groups: WC indicates Wistar Control (n = 8), WCT indicates Wistar Control Tumor (n = 8), and WTT indicates Wistar Trained Tumor (n = 8). The results expressed in grams (g) are the weight of the tumor, the body weight, and the tumor-free body weight. Tumor volume is expressed in cubic centimeters (cm^3^) and percentage of body weight loss (%). Statistical analysis: One-way ANOVA, Tukey’s *post hoc* test. Significant different vs. $ indicates the weekly difference in body weight for all groups, p < 0.0001. *WC, p < 0.05; **WC, p < 0.01; ***WC, p < 0.001; ****WC, p < 0.0001; ^†^WCT, p < 0.05; ^‡^WCT, P < 0.01; ^††^WCT, p < 0.001; ^‡‡^WCT, p < 0.0001. Signicant different vs. # indicates the difference between pre-test and post-test. #p < 0.05; ##p < 0.001, ### p < 0.001; #### p < 0.0001.

**Figure 2A** shows the animals’ BW increased exponentially in the measurements carried out between the period day -25 to day 21 in both WC and WTT groups. In contrast, the WCT group exhibited a reduction in the rate of weekly weight gain over the same period. In **Figure 2B**, the final BW without tumor was analyzed, indicating a decrease in the WCT group (297.88 ± 13.06 g; p < 0.0001) compared to the WC group (357.35 ± 9.26 g). Similar results are found in the WTT group (263.54 ± 8.26 g; p <0.0001) compared to the WC group. There was no difference between the groups WTT and WCT (p < 0.07).

The tumor volume of the WCT group increased since its second measurement on day 6 (p < 0.01), showing a statistical difference until the last day 18 (p < 0.05). As observed in the WCT group, the WTT group showed a significant increase from the second measurement on day 6 (p < 0.05), followed by a developmental delay starting on day 13 (p < 0.001) (**Figure 2C**). When the WCT and WTT groups were compared, the tumor development was different from its measurement on day 6 (p < 0.01) until the last day 18 (p < 0.01), thus evidencing a greater evolution of the WCT group in relation to the group tumor trained. The WTT group showed a reduction in tumor weight (25.08 ± 4.1 g; p < 0.001) (**Figure 1D**) and in the percentage of cachexia (7.83 ± 1.75%; p < 0.05) (**Figure 1E**) compared to the WCT group (47.08 ± 6.0 g; 13.75 ± 1.71%; respectively), indicating that AET retarded tumor growth and cancer cachexia in Walker 256 tumor-bearing rats.

### Effect of AET on skeletal muscle wasting of walker-256 tumor-bearing rats

**Figure 3:**
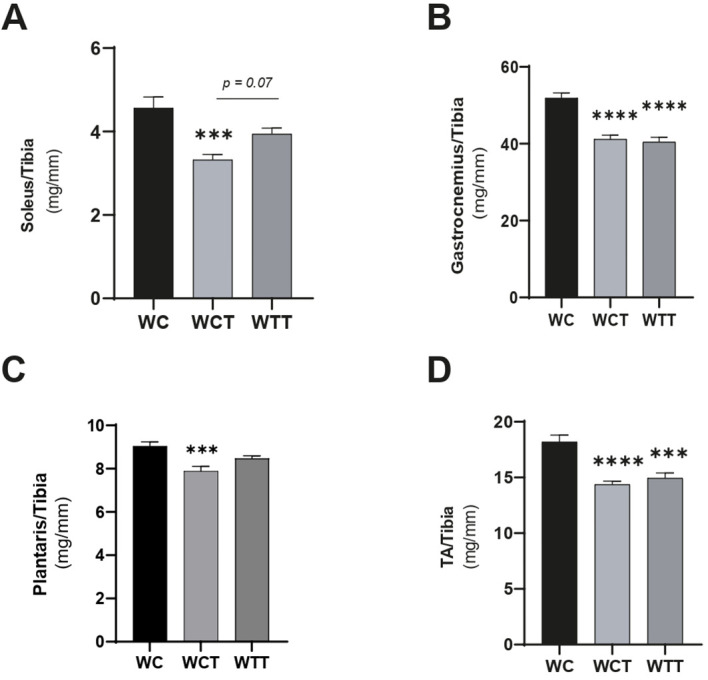
Aerobic exercise training prevents muscle wasting induced by Walker-256 cancer. Soleus weight corrected for tibial length **(A)**, gastrocnemius weight corrected for tibial length **(B)**, plantar weight corrected for tibial length **(C)**, and tibialis anterior weight corrected for tibial length **(D)**. Groups: WC indicates Wistar Control (n = 8), WCT indicates Wistar Control Tumor (n = 8), and WTT indicates Wistar Trained Tumor (n = 8). Results are expressed as mean ± SEM. Statistical analysis: One-way ANOVA, Tukey’s *post hoc* test. Significant different vs. ***WC, p < 0.001; ****WC, p < 0.0001.

Regardless of skeletal muscle mass, a significant muscle wasting was observed in the all dissected muscles, with a reduction in soleus (3.32 ± 0.13 mg/mm; p < 0.001), gastrocnemius (39.31 ± 2.08 mg/mm; p < 0.001), plantaris 7.89 ± 0.22; p < 0.001), and TA muscle (13.83 ± 0.61; p < 0.0001) in the WCT group compared to the WC group (4.56 ± 0.26 mg/mm; 51.86 ± 1.35 mg/mm; 9.03 ± 0.20 mg/mm; 18.19 ± 0.61 mg/mm; respectively) (**Figures 3A–D**, respectively). On the other hand, soleus and plantaris muscle mass did not differ between the WC group (4.56 ± 0.26 mg/mm and 9.03 ± 0.20 mg/mm, respectively) and WTT group (3.94 ± 0.14 mg/mm and 8.48 ± 0.12 mg/mm, respectively), whereas both were reduced in the WCT group (**Figures 3A, C**, respectively). However, gastrocnemius and tibialis anterior muscle mass remained lower in the WTT group compared with the WC group.

### Effect of AET on tissues weight of walker-256 tumor-bearing rats

Lung weight was significantly increased in the WCT group compared to the WC group (p < 0.01). In contrast, the WTT group exhibited a reduction in lung weight compared to WCT (p > 0.01), although it remained different from WC (p > 0.05). A similar pattern was observed for liver weight, which was significantly elevated in the WCT group compared to WC (p < 0.05). However, liver weight in the WTT group was markedly reduced compared to WCT (p < 0.0001) and showed no significant difference relative to WC. Spleen weight was also significantly increased in the WCT group compared to WC (p < 0.0001), but was normalized by AET in the WTT group. Regarding kidney weight, the WTT group showed a significant reduction compared to both WC (p < 0.01) and WCT (p < 0.05) (**Table 2**). Additionally, AET led to a significant reduction in retroperitoneal and periepididymal fat weight in the WTT group when compared to both WC (p < 0.0001) and WCT (p < 0.01) (**Table 2**). The reduction in fat depots was only observed in the WTT, and not in the WCT group.

**Table 2:** Tissues.

Tissues	WC	WCT	WTT
Lung (mg/mm)	44.97 ± 1.94	64.41 ± 5.35**	53.97 ± 2.20*†
Liver (mg/mm)	345.61 ± 14.39	362.91 ± 20.01*	297.61 ± 10.01‡‡
Kidney (mg/mm)	72.21 ± 2.82	69.62 ± 2.82	59.23 ± 1.91**†
Spleen (mg/mm)	18.45 ± 0.70	58.19 ± 8.18****	34.54 ± 2.12‡
Retroperitoneal Fat (mg/mm)	123.63 ± 13.02	93.25 ± 9	47.91 ± 5.41****‡
Periepididymal Fat (mg/mm)	111.94 ± 7.99	100.14 ± 6.87	51.02 ± 4.87****††

Groups: WC indicates Wistar Control (n = 8); WCT Wistar Control Tumor (n = 8) and WTT, Wistar Trained Tumor (n = 8). Results are expressed as mean ± SEM. Statistical analysis: One-way ANOVA, Tukey’s post hoc test. Significant different vs. *WC, p < 0.05; **WC, p < 0.01; ****WC, p < 0.0001; ^†^WCT, p < 0.05; ^‡^WCT, P < 0.01; ^††^WCT, p < 0.001; ^‡‡^WCT, p < 0.0001.

### Effect of AET on cardiac remodeling of walker-256 tumor-bearing rats

**Figure 4:**
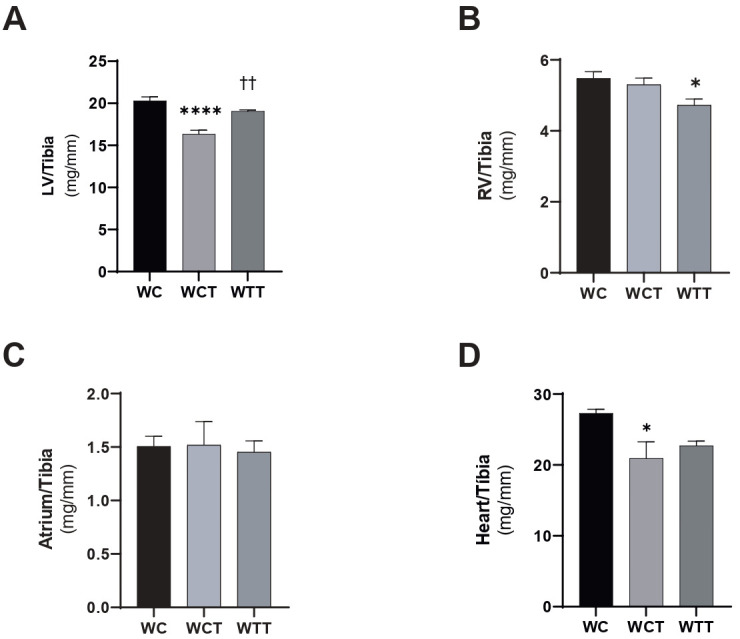
Aerobic exercise training prevents heart atrophy induced by Walker-256 cancer. Left ventricle weight corrected for tibial length **(A)**, right ventricle weight corrected for tibial length **(B)**, atrium weight corrected for tibial length **(C)**, and total heart weight corrected for tibial length **(D)**. Groups: WC indicates Wistar Control (n = 8), WCT indicates Wistar Control Tumor (n = 8), and WTT indicates Wistar Trained Tumor (n = 8). Results are expressed as mean ± SEM. Statistical analysis: One-way ANOVA, Tukey’s *post hoc* test. Significant different vs. *WC, p < 0.05; ****WC, p < 0.0001. ^††^WCT, p < 0.001.

The LV weight (**Figure 4A**) showed a reduction in the WCT group (15.38 ± 1.05 mg/mm; p < 0.0001) compared to the WC group (20.30 ± 0.47 mg/mm). However, AET increased the LV mass in the WTT group (19.05 ± 0.66 mg/mm; p < 0.001) when compared to the WCT group. The RV weight (**Figure 4B**) showed a reduction in the WTT group (4.72 ± 0.18 mg/mm; p < 0.05) compared to the WC group (5.48 ± 0.19 mg/mm). For the atrium weight (**Figure 4C**), there was no statistical difference between the groups. Like the results found in LV mass, the total heart weight (**Figure 4D**) showed a reduction in the WCT group (20.93 ± 2.32 mg/mm; p < 0.01) compared to the WC group (27.28 ± 0.57 mg/mm). However, heart weight in the WTT group (22.72 ± 0.64 mg/mm) was higher than in the WCT group (p < 0.05).

### Effect of AET on morphometric parameters by echocardiogram of walker-256 tumor-bearing rats

**Figure 5:**
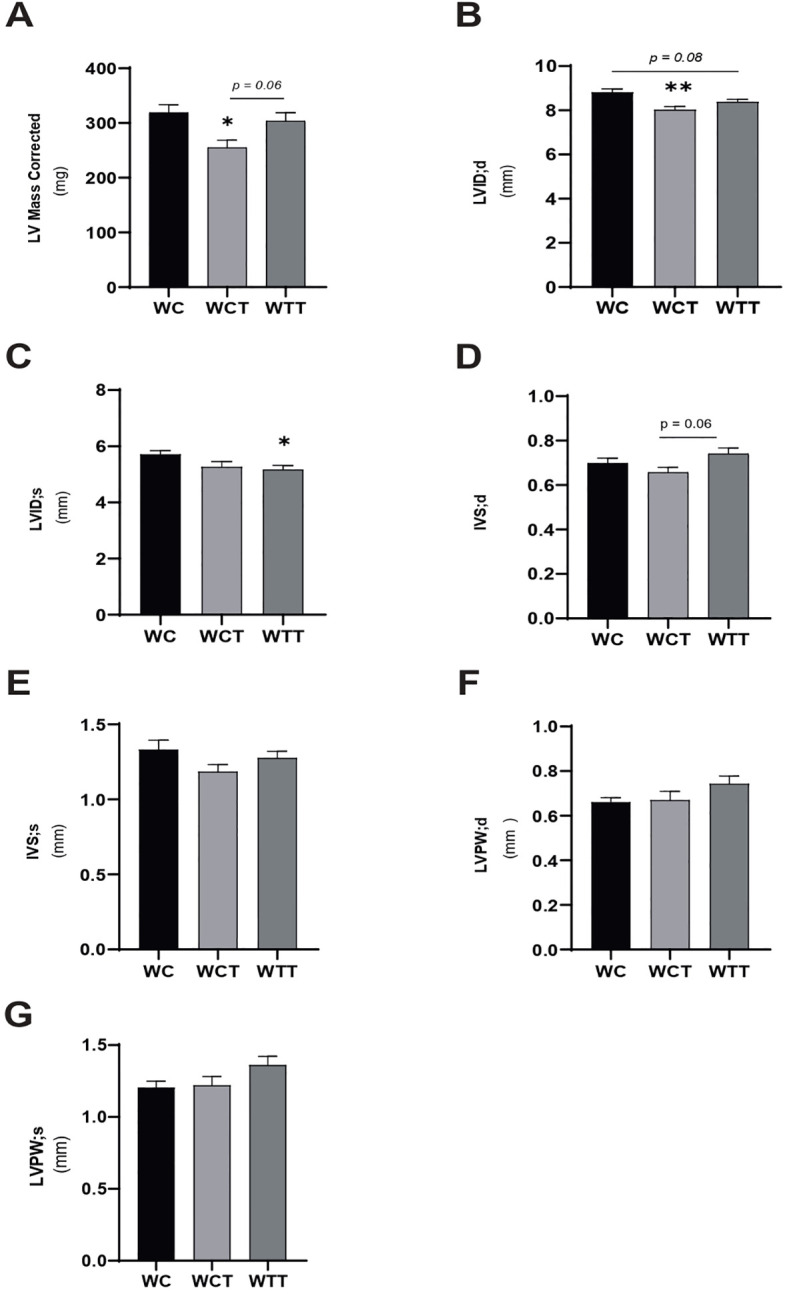
Aerobic exercise training prevents cardiac remodeling induced by Walker-256 cancer, assessed by Echocardiographic measurements. Left ventricle mass (LV Mass Corrected) **(A)**, left ventricle internal diameter in diastole (LVID;d) **(B)**, left ventricle internal diameter in systole (LVID;s) **(C)**, interventricular septal thickness in diastole (IVS;d) **(D)**, interventricular septal thickness in systole (IVS;s) **(E)**, thickness of the left ventricular posterior wall in diastole (LVPW;d) **(F)**, and thickness of the left ventricular posterior wall in systole (LVPW;s) **(G)**. Groups: WC indicates Wistar Control (n = 8), WCT indicates Wistar Control Tumor (n = 8), and WTT indicates Wistar Trained Tumor (n = 8). Results are expressed as mean ± SEM. Statistical analysis: One-way ANOVA, Tukey’s *post hoc* test. Significant different vs. *WC, p < 0.05; **WC, p < 0.01.

Corroborating the findings observed in the wet weight of the LV and heart, the LV mass measured by echocardiogram was significantly reduced in the WCT group (254.77 ± 13.59 mg) compared to the WC group (318.92 ± 14.63 mg; p < 0.05) (**Figure 5A**). In contrast, AET increased cardiac mass in the WTT group (303.50 ± 15.42 mg) compared to the WCT group. The LVID;d was significantly reduced in the WCT group (8.02 ± 0.13 mm; p < 0.01) compared to the WC group (8.81 ± 0.15 mm) (**Figure 5B**). This reduction in LVID;d was prevented by AET in the WTT group (8.23 ± 0.17 mm; p > 0.05) compared to the WC group (**Figure 5B**). Regarding the LVID;s, a small but statistically significant difference was observed between the WTT (5.17 ± 0.14 mm) and WC (5.26 ± 0,18 mm) (**Figure 5C**). No significant differences were found among groups in the IVS;s and IVS;d, nor in the LVPW;d and LVPW;s as shown in **Figures 5E–G**.

### Effect of AET on cardiac function by echocardiogram of walker-256 tumor-bearing rats

In the echocardiographic function analyses, MV E increased in the WTT group (p < 0.01) compared to the WCT group. No difference was observed between the WC and WTT groups. The other parameters analyzed in **Table 3** did not show statistical difference.

**Table 3:** Cardiac function by echocardiogram.

Cardiac function	WC	WCT	WTT
EF (%)	62.2 ± 1.2	61.1 ± 2.8	62 ± 1.2
FS (%)	35.1 ± 0.9	34.3 ± 2	37.1 ± 1.3
CO (ml/min)	256.4 ± 9.8	231.6 ± 11.69	256 ± 9.8
MV A (mm/s)	644.1 ± 43.8	709.4 ± 40.3	750.2 ± 64.1
MV E (mm/s)	1011.7 ± 23.7	951 ± 29.5	1070.3 ± 24.2‡
MV Decel (ms)	18.3 ± 1.2	14.5 ± 0.9	19.2 ± 2.4
MV E/A	1.6 ± 0.1	1.3 ± 0.05	1.5 ± 0.1
MVET (ms)	45.9 ± 2.1	49.9 ± 2.2	53.2 ± 3.8
IVRT (ms)	20.2 ± 0.6	22.5 ± 1.7	20.5 ± 0.6

Ejection fraction (EF), Shortening fraction (FS), Cardiac output (CO), A wave (MV A), E wave (MV E), E wave deceleration (MV Decel), E/A ratio, ejection time (MV ET), and isovolumetric relaxation time (IVRT). Groups: WC indicates Wistar Control (n = 8); WCT Wistar Control Tumor (n = 8); and WTT, Wistar Trained Tumor (n = 8). Results are expressed as mean ± SEM. Statistical analysis: One-way ANOVA, Tukey’s post hoc test. **WC, p < 0.01; ‡WCT, p < 0.01.

## Discussion

AET is widely recognized for its preventive and therapeutic efficacy in chronic non-communicable diseases, contributing to mitigate the cachexia status and the pathological cardiac anti-remodeling (9–11, 18, 32). Our study showed AET was able to reduce cancer cachexia in the animals. The results found are like those presented in the literature (22, 23). In addition, AET delayed the tumor progression, consequently the tumor weight was lower in trained animals. The weight of the animals showed exponential progression among the groups, but when the tumor-free weights were analyzed, lower weights were found for the groups with Walker- 256 tumor. A possible explanation for the weight differences observed in the tumor-bearing groups is the reduction in body fats in the trained group (**Table 2**). The AET-induced fat loss has been consistently reported in several studies (22, 23). Additionally, the weights of other organs, such as kidneys, liver, spleen and lungs, also reflected the overall differences in BW between the tumor-bearing groups, further supporting this finding.

The Walker-256 tumor promotes three carcinogenesis stages: initiation, promotion and progression in a brief period of 12–16 days. It has accelerated growth causing cachexia, like what occurs with cancer patients (38–40). Since the tumor growth period is short, lasting no more than 21 days before euthanasia, this exercise training period would be insufficient to achieve cardiovascular adaptations resulting from AET (38–40). Our group has studied the effects of AET on cardiovascular adaptations and physical performance, and we observed that a period of less than six weeks would be inadequate to achieve these benefits (11, 38–40). Therefore, three weeks of prior training were necessary, followed by an additional three weeks concomitantly with inoculation and tumor growth to achieve both the beneficial effects of AET and the tumor growth time for cachexia induction (11, 38–40).

The spleen is the largest immune organ in the human body. Its abnormal growth of the spleen, seen in the WCT group, is known as splenomegaly. This pathology can give rise to complications due to the reduction in the number of red blood cells, white blood cells and platelets in the bloodstream, making the organism more susceptible to infections, anemias and bleeding (38). It can be characterized in three ways: increased splenic function, infiltration or congestion. Malignant, benign or metabolic conditions can also lead to splenomegaly (38). Exercise has been shown to prevent this increase in the WTT group, highlighting the potential of AET, such as therapy for reductions in structural data caused by cancer (38–40).

When we analyzed the exercise tolerance, performed in the exercise test, the WCT group showed a reduction in both running distance and time compared to their own pre-test performance. Furthermore, in the post-test, the WCT group exhibited the shortest running distance and duration among all groups. These findings indicate that tumor progression was a determining factor contributing to exercise intolerance. On the other hand, the trained group showed improvement in results compared to its pre-test and improvement compared to the WCT group in the post-test. In relation to muscle mass, AET preserved soleus and plantaris muscle mass in tumor bearing rats, whereas gastrocnemius and tibialis anterior mass remained reduced compared with controls, indicating a partial and muscle specific effect of training on cachexia induced muscle wasting. In a study carried out by Cella et al. (39), a reduction in the transversal area of ​​some muscles was demonstrated and the cause for this reduction was splenomegaly, as it triggers an inflammatory and catabolic state facilitating cachexia. Additionally, AET retarded cancer cachexia in Walker-256 tumor-bearing rats by a decrease in skeletal muscle degradation process associated with an increase in skeletal muscle myosin content (34).

The LV mass was reduced in the cancer group compared to the control; however, when we look at the RV, only the animals with Walker-256 tumor trained group shows a reduction. A study by Borges et al. (40) showed a reduction in the RV of animals with Walker-256 tumor, similar to that found in our study. For the first time, we observed that AET prevented Walker-256 tumor-induced cardiac atrophy in trained animals, as assessed by heart weight and echocardiographic measurements. It is possible that part of the cardiac protection observed in the WTT group is mediated by the reduction in tumor burden and its systemic consequences, and our data do not allow us to distinguish myocardium-specific training effects from those related to tumor reduction. Similar results induced by AET were described in other cancer models. A recent study of Fernandes et al. (11) observed that although AET was not able to counteract the reduction of heart mass in Colon cancer, a partial effect was observed in cardiomyocyte diameter and EF, since no differences were observed between cancer trained and healthy control mice. Thus, the authors concluded that although AET promoted an increase in cardiomyocyte diameter, it was also accompanied by a reduction in collagen deposition, which impacted the reduction in the final weight of the heart in the trained cancer group (11). Our echocardiogram data indicated that the LVID;d was reduced in the cancer group compared to the control group, suggesting a reduction in the measurement of the size of the LV at its widest point, just before it starts to contract. However, AET was able to prevent this reduction. In addition, the increase in E wave in the WTT group compared to the WCT group may be an attempt to compensate for excess blood volume in the heart, bringing values like those of the control group.

In summary, our data show that animals with Walker-256 cancer developed cachexia characterized by loss of muscle mass and tumor-free body weight. These changes were accompanied by cardiac atrophy, which jointly contributes to reduced aerobic capacity. On the other hand, the AET was able to promote beneficial changes and reduce the damage caused by disease. This is the first study that proposed to study the role of AET in changes in the cardiac phenotype promoted by the Walker-256 tumor. Our design mimics patients who engage in regular aerobic exercise before diagnosis or before starting aggressive cancer treatments (e.g., chemotherapy, major surgery, or radiotherapy), thereby entering treatment with a better cardiorespiratory fitness reserve, muscle mass, and cardiovascular resilience. Clinical and translational data indicate that higher pre-treatment fitness and muscle mass are associated with better tolerance to therapy, fewer complications, and improved prognosis in cancer patients, which conceptually parallels the “preconditioned” status of WTT animals in our model (41, 42). Therefore, our findings support the idea that exercise prehabilitation—initiated before or as early as possible in the cancer trajectory—may help attenuate treatment and disease related functional decline, cachexia, and cardiac damage. Despite this translational parallel, the Walker−256 model has a much shorter time course than human cancer, and that future clinical studies are needed to determine the optimal type, timing, and duration of prehabilitation programs in patients with different tumor types and treatment regimens.

A limitation of the present study is the absence of a healthy trained group. Including such a group would have allowed us to define the maximal physiological and cardiac adaptations to the AET protocol in healthy rats. Nevertheless, exercise induced cardiovascular and skeletal muscle adaptations in healthy animals are well characterized and do not mirror the pathophysiological milieu of tumor bearing rats. Due to ethical and logistical constraints on animal number, we prioritized groups that directly addressed our primary aim (sedentary control vs. sedentary tumor vs. trained tumor). In addition, molecular data may help explain the observed physiological and morphological changes, which is considered a limitation of the study. As a perspective, molecular analyses of markers of apoptosis, inflammation, and oxidative stress in cardiac and skeletal muscle damage need to be performed to better understand these adaptations promoted by cancer and exercise.

## Data Availability

The original contributions presented in the study are included in the article/supplementary material. Further inquiries can be directed to the corresponding author.
